# Functional Anatomy of the Rear Attachment of the Deep Deltoid (RAD) Ligament of the Ankle

**DOI:** 10.7759/cureus.60442

**Published:** 2024-05-16

**Authors:** Radwane Faroug, Suheil Amanat, Benjamin Schapira, Aashish Raghu, Michael Tombros

**Affiliations:** 1 Trauma and Orthopaedics, Stoke Mandeville Hospital, Buckinghamshire Healthcare NHS Trust, Milton Keynes, GBR; 2 Trauma and Orthopaedics, Oxford University Hospitals NHS Foundation Trust, Oxford, GBR; 3 Trauma and Orthopaedics, Bedfordshire Hospitals NHS Foundation Trust, Luton, GBR; 4 Trauma and Orthopaedics, Lister Hospital, Stevenage, GBR; 5 Trauma and Orthopaedics, Forte Sports, Christchurch, NZL

**Keywords:** rear attachment deep deltoid ligament, ankle stability, ankle injury, deltoid ligament, deep deltoid ligament

## Abstract

The deltoid ligament plays a key role in ankle stability. Ankle fractures involving the medial ligamentous complex are evaluated on the basis of medial-sided tenderness and the Ottawa ankle rules. Evolution in our understanding of this ligament over the last three decades has shown that, within this medial ligamentous complex, it is the deep deltoid ligament that confers mechanical stability. The latest evolution in this understanding, and the learning point of this report, is that only a distinct component of the deep deltoid ligament - specifically the discreet posterior third - the rear attachment of the deep deltoid ligament (RAD) - confers mechanical value. The RAD is responsible for providing the medial ligamentous component of ankle stability - specifically talar shift, tilt, and importantly rotational stability. This knowledge is of key importance in the assessment and management of ankle fractures with associated deltoid ligament injuries. In this technical report, we highlight the biomechanical contribution of the RAD, which will help surgeons and physiotherapists to accurately manage ankle injuries.

## Introduction

Ankle injuries such as sprains and fractures are one of the most common presentations to the emergency department [1]. Patients with suspected injuries are commonly evaluated clinically via the Ottawa Ankle Rules prior to undertaking radiographs to exclude unstable ankle fractures. This is an accepted tool comprised of four criteria - bony tenderness along the distal 6 cm of the posterior edge of the tibia or medial malleolus; bony tenderness along the distal 6 cm of the posterior edge of the fibula or lateral malleolus tip; and inability to bear weight both immediately and for four steps in the emergency department [2].

The presence of any of these findings is an indication of an ankle X-ray [2]. The presence of tenderness over the tip of the medial malleolus broadly signifies deltoid ligament injury or medial malleolus fracture. Further classifications of ankle injuries include the Lauge-Hansen criteria, which classify injuries based on mechanism and foot position at the time of injury, providing predictable injury patterns and radiographic findings. Classically, this medial tenderness has been interpreted as equating to an unstable ankle fracture. However, this does not specifically indicate injury to the deep deltoid ligament component, and, specifically, the RAD, which we now understand, is of critical importance in determining ankle stability, particularly in Lauge-Hansen type IV supination-external rotation (SER) injury [3].

In this technical report, we highlight the current and latest evidence outlining the importance of deep deltoid ligament injury in causing unstable ankle fracture injuries, where instability may persist following standard surgical fixation of bi-malleolar ankle fractures. If the deep deltoid injury is not recognized, specifically to the RAD, there is a potential risk of significant impact on rehabilitation, mobilization, and clinical outcome.

## Technical report

The evolution in understanding the functional role of the deltoid ligament has progressed through multiple stages. Firstly, it was believed that medial tenderness on palpation with or without ecchymosis was associated with ankle instability which would require fixation. 

**Figure 1:**
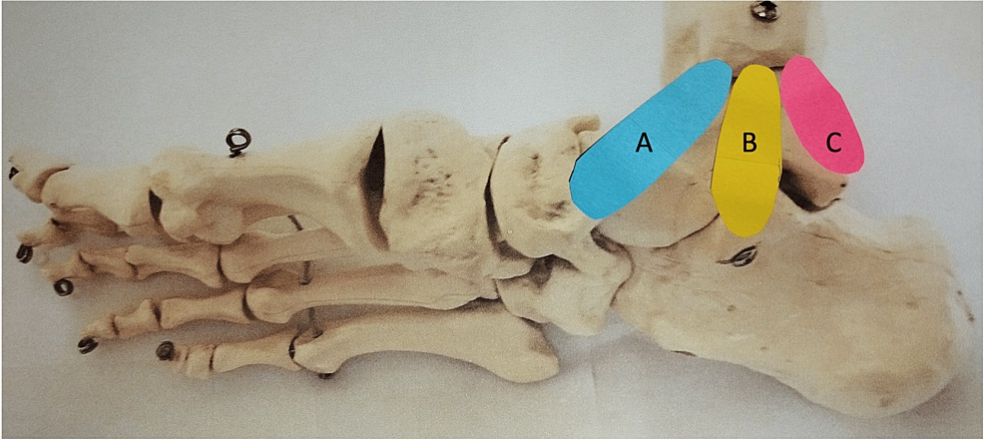
Three-leaf fan-like arrangement of the deep deltoid component ligament An anatomical model demonstrating the three-leaf fan-like arrangement of the deep deltoid component ligament. (A) represents the deep anterior tibia-talar ligament (anterior deep deltoid), (B) represents the deep intermediate tibio-talar ligament (middle deep deltoid), and (C) represents the deep posterior tibio-talar ligament or rear attachment of the deep deltoid (RAD).

The deltoid ligament can be split into superficial and deep components, of which it has been found that the deep, rather than superficial, strata confer mechanical stability. The deep component has a specific and reproducible arrangement of fibers in a distinct three-leaf fan-like pattern consisting of the deep anterior tibio-talar ligament, deep intermediate tibio-talar ligament, and the posterior leaf - the deep posterior tibio-talar ligament [4] (Figure 1).

**Figure 2:**
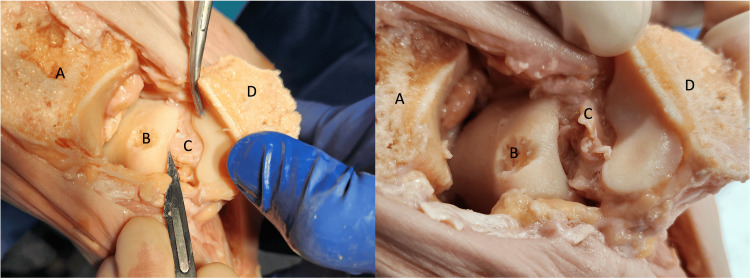
Cadaveric photograph showing the origin and insertion of the RAD. Cadaveric photograph showing the origin and insertion of the RAD originating from the posterior colliculus of the medial malleolus and inserting on the supero-postero-medial talar tubercle. (A) represents the osteotomized edge of the medial tibia, (B) talus, (C) RAD, and (D) osteotomised and reflected medial malleolus. Permission was granted for the use of cadaveric images from POZNAŃ LAB (Institute of Practical Medicine, Poznań, Poland).

**Figure 3:**
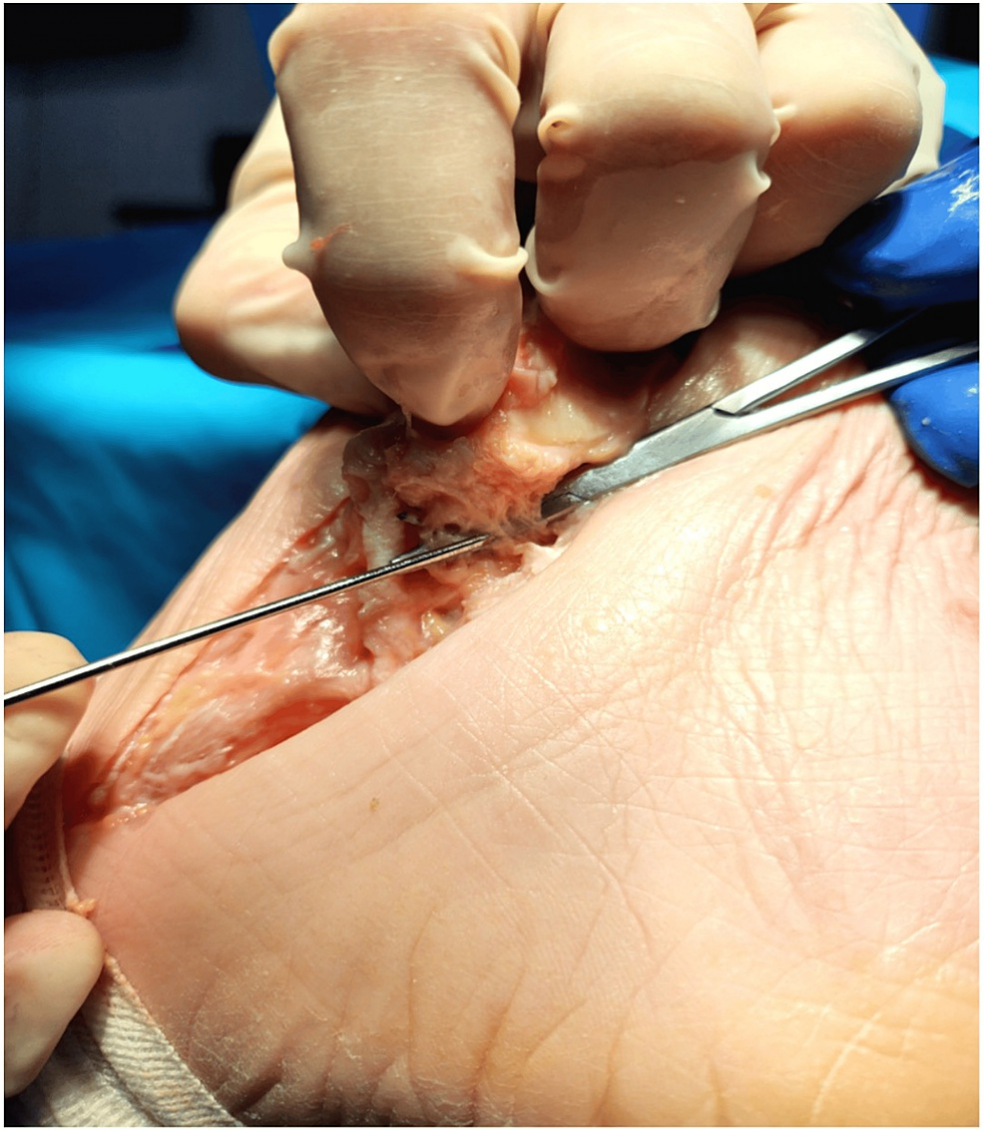
Cadaveric photograph of an osteotomised and flipped medial malleolus demonstrating the discreet fibers of the RAD. A cadaveric photograph of an osteotomised and flipped medial malleolus demonstrating the discreet fibers of the RAD. Permission was granted for the use of cadaveric images from POZNAŃ LAB (Institute of Practical Medicine, Poznań, Poland).

Further anatomical and cadaveric biomechanical studies demonstrated that it was specifically the posterior third fibers or RAD that provide this stability in relation to talar shift, tilt, and rotation [5]. This ligament originates from the posterior colliculus of the medial malleolus and is inserted into the supero-postero-medial talar tubercle [6] (Figures 2-3).

From cadaveric anatomical dissection, we have found the RAD to be discreet in nature as a clearly definable ligament and warrants specific focus in the repair of the deep deltoid ligament. This anatomical study highlights the mechanically relevant portion of the deep deltoid ligament.

## Discussion

A typical pattern of injury occurs in an SER ankle injury [3]. When the ankle is positioned in supination and is subjected to an external rotation force, this causes a circular pattern of injury initially to the lateral ankle structures and finally involves the medial structures. Firstly, the antero-inferior tibio-fibular ligament (AITFL) is torn (SER I). Continued external rotation causes a short-oblique lateral malleolus fracture (SER II), postero-inferior tibio-fibular ligament (PITFL) tear, or posterior malleolar fracture (SER III), followed by deltoid ligament injury or medial malleolar fracture (SER IV). This technical report focuses on type IV injuries involving the deltoid ligament, specifically the discreet fibers of the RAD.

Studies have demonstrated a poor association between general medial ankle tenderness and deltoid ligament injury [7,8]. Furthermore, Lauge-Hansen’s classification is based on the simulation of mechanical injury to cadaveric ankle specimens, which can differ from clinical injury mechanisms and patterns in patients.

The traditional radiographic evidence of deltoid ligament injury, and ankle instability, is the presence of a bi-malleolar bony injury, increased medial clear space (>4 mm), and lateral talar shift on weight-bearing radiographs. However, these signs are of poor significance in assessing ankle stability as per Lauge-Hansen’s modified classification (SER IV a and b), because stable variants may in fact be associated with deep deltoid ligament injury (SER IV a) [9]. Furthermore, Chhabra et al. in their MRI study noted that deep deltoid ligament injuries were more frequent than superficial deltoid ligament injuries [10].

A recent cadaveric biomechanical study [5], evaluating the discreet ankle ligament components, used eight cadaveric ankle specimens, which were stressed by axial loading and external rotation using a custom rig. The individual ankle ligaments were sectioned using a standardized technique sequentially as per a typical supination-external rotation injury pattern (AITFL, PITFL, superficial deltoid, deep deltoid anterior third, middle third, and posterior third). It was noted that ankle instability in the form of increased medial clear space (>4 mm) and increased talar tilt (>7°) appeared only at the point of sectioning the RAD ligament. Ankle instability, as defined above, did not occur on dividing the anterior and intermediate deep deltoid ligaments with an intact RAD.

This study thus draws attention to the relevance of the individual and discreet ligamentous components of the deep deltoid ligament and their potential role in ankle instability within the context of their anatomical position.

## Conclusions

This new evidence underscores the latest understanding of the functional anatomy of the deep deltoid ligament of the ankle. It focuses on the discreet role of the RAD in determining ankle stability or instability and impacts on both clinical and surgical decision-making in managing ankle SER IV injuries. Surgically, ankle fractures with an associated RAD injury may need deep deltoid reconstruction to address any persisting talar tilt after adequate bony fixation.
